# Supplementary figures and images for: Transplantation of human iPSC-derived muscle stem cells in the diaphragm of Duchenne muscular dystrophy model mice
Source: PLoS One. 2022 Apr 4;17(4):e0266391. doi: 10.1371/journal.pone.0266391 (PMC8979463; doi:10.1371/journal.pone.0266391)

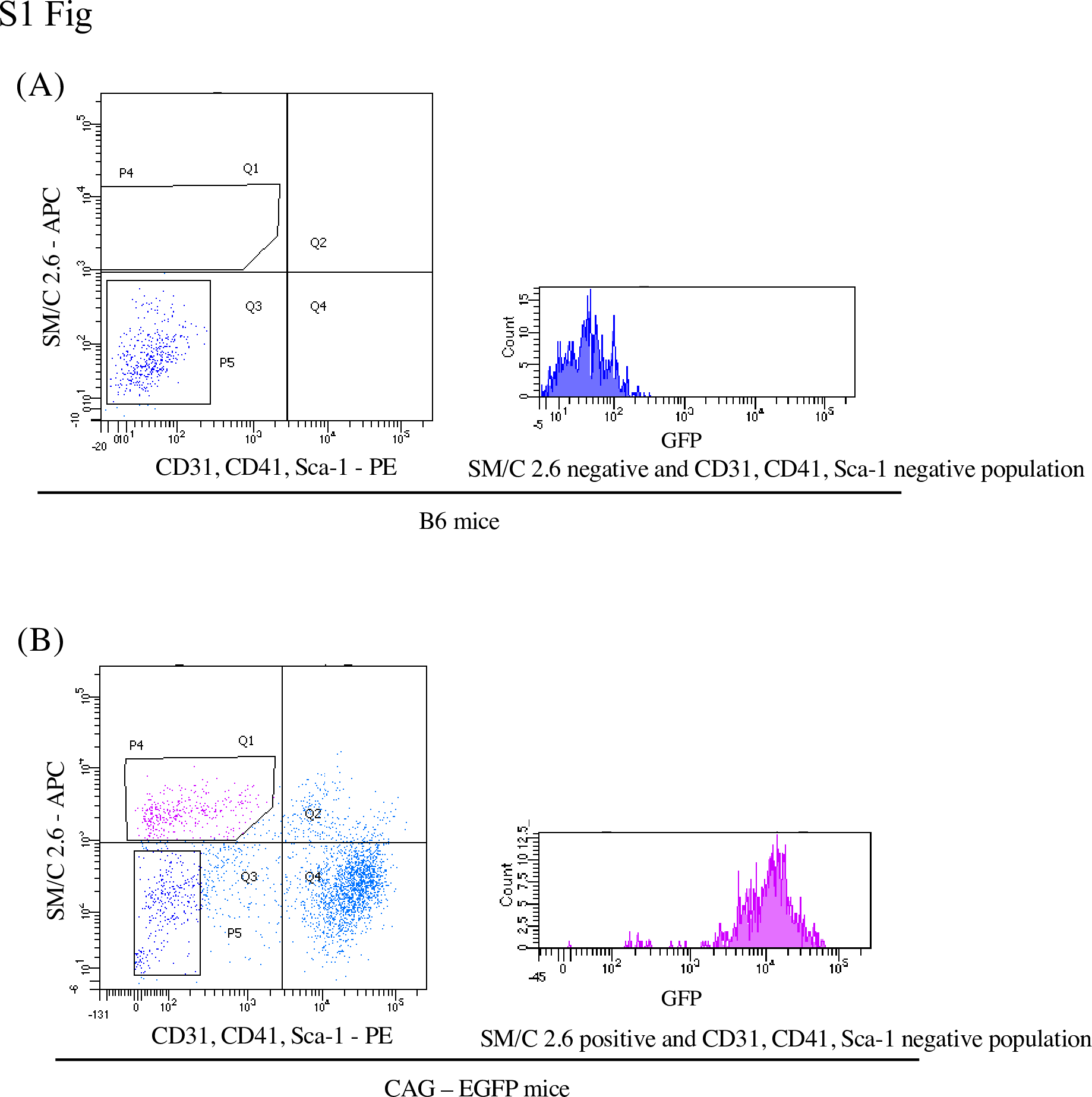

Supplement: S1 Fig — (A) FCM analysis of isolated cells from B6 mice (left panel) and GFP expression (right panel). (B) FCM analysis of isolated primary satellite cells from CAG-EGFP mice (left panel) and GFP expression (right panel). (TIF) [file pone.0266391.s001.tif]

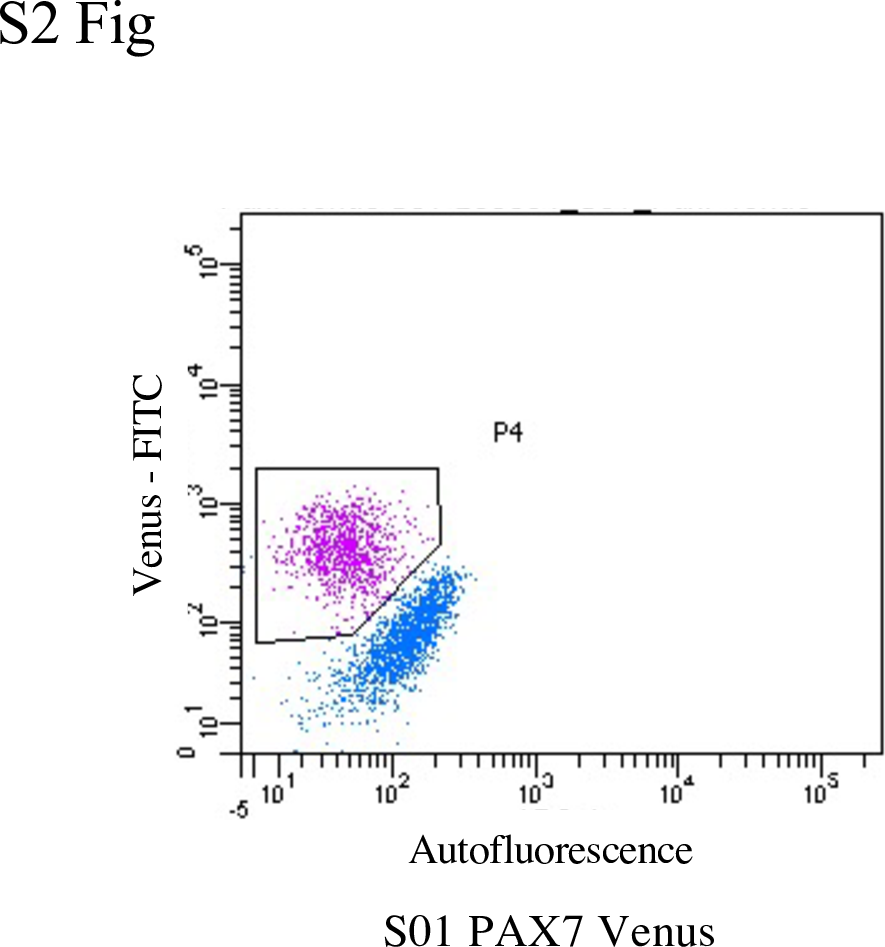

Supplement: S2 Fig — (TIF) [file pone.0266391.s002.tif]

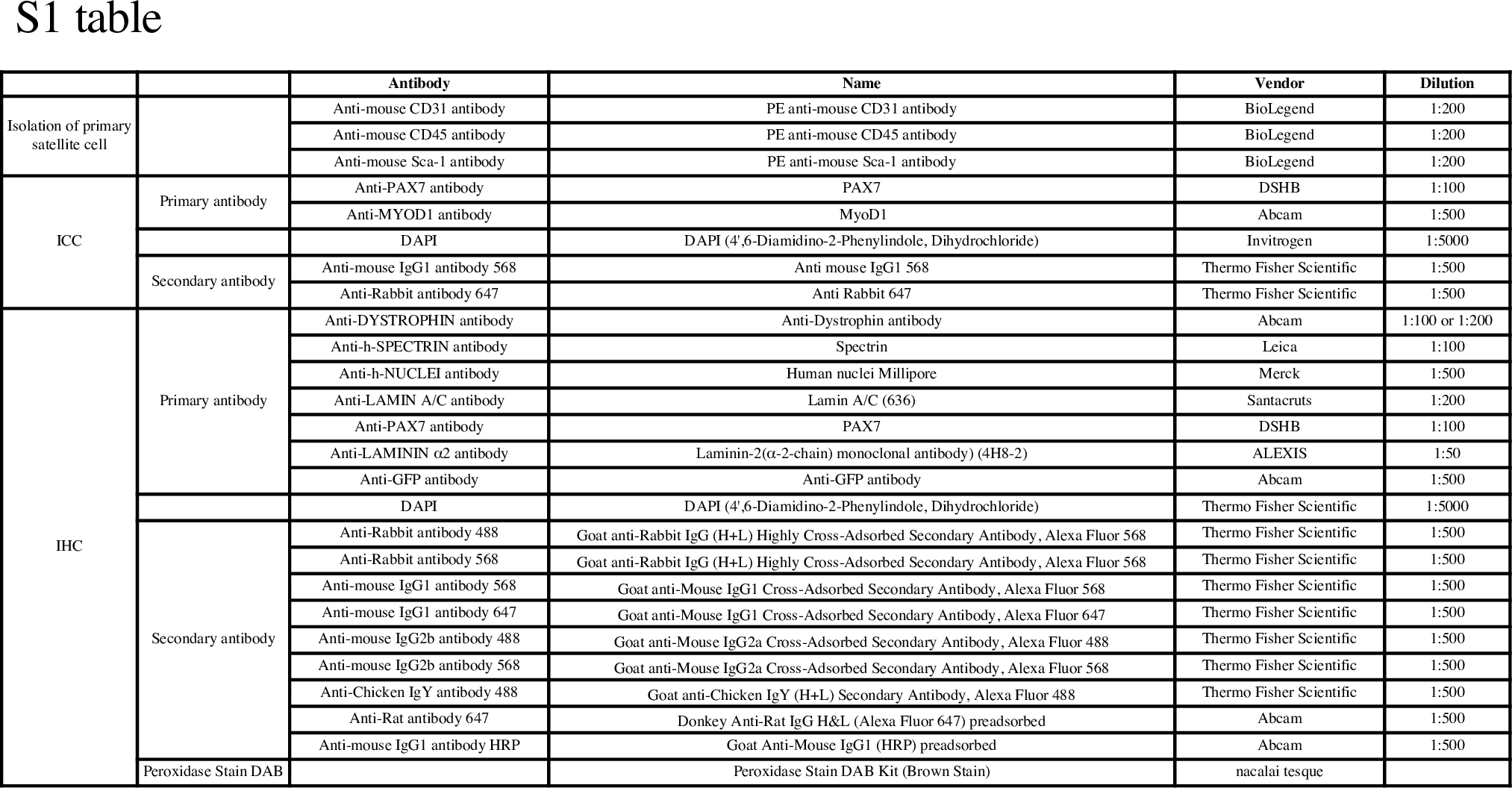

Supplement: S1 Table — (TIF) [file pone.0266391.s003.tif]
