# Supplementary material for: Transplantation of human iPSC-derived muscle stem cells in the diaphragm of Duchenne muscular dystrophy model mice
Source: PLoS One. 2022 Apr 4;17(4):e0266391. doi: 10.1371/journal.pone.0266391 (PMC8979463; doi:10.1371/journal.pone.0266391)
Supplement: Attachment — Submitted filename: response_to_comment_R2_ver2.docx [file pone.0266391.s005.docx]

Editor comment:

1. the discrepancy of GFP expression and DYSTROPHIN expression observed in figure 2 (including the nuclear domain of Dystrophin);

A：We are considering two reasons for why the number of DYSTROPHIN positive fibers was less than that of GFP positive fibers in the Hu5/KD3 transplantation. One possibility for the discrepancy is the different expression levels of GFP and DYSTROPHIN. GFP expression was promoted by the strong EF1a promotor in the piggyBac vector, which was multiply transduced, while DYSTROPHIN expression was promoted using an endogenous DYSTROPHIN promotor. The other is that 4 weeks after the transplantation might not be long enough to see DYSTROPHIN positive fibers in the Hu5/KD3 transplantation.

Furthermore, we reanalyzed GFP positive fibers per GFP positive area in the serial sections of the samples shown in Fig. 1 and Fig. 2 (Fig. R1 below) and found a smaller signal than with the transplantation of mouse primary cells.

Fig. R1 Quantification of GFP positive fibers in the transplanted areas. Data show the mean ± SEM (two-tailed Student’s t-test; ****p<0.0001) and are from three independent experiments. Three mice were used in one experiment. There were a total of 59 primary satellite cells and 36 GFP transgenic Hu5/KD3 cells in the respective GFP areas.

2. The use of human-specific SPECTRIN Abs instead of DSYTROPHIN Abs;

A：The signal from the DYSTROPHIN antibody was weaker than the signal from the h-SPECTRIN antibody. Therefore, we counted h-SPECTRIN positive fibers as myofibers derived from engrafted cells. We added new Fig. 3B to show these observations and explained the reason why we used h-SPECTRIN antibody in the revised text (Page 18, line 298).

3. To amend the title of figure 4 and the general discussion / conclusion of the article to better reflect the results obtained (going from 1 to 4 spectrin-positive fibers remains very low).

A：As suggested, we changed the title of the manuscript “H2G8 reduces the frequency of iMuSC engraftment failure in the diaphragm of NOG-mdx mice” to and modified the general discussion.

#2 reviewer comment:

Unfortunately, the revised manuscript still falls short in convincing the reader that meaningful (real) engraftment has been reached with human cells. How can one refer to regenerative potential when transplantation of Hu5/KD3 cells only shows one green fiber (which is not even co-localized with dystrophin)? The authors did not address my concern regarding iMusc transplantation. The explanation provided for not using dystrophin staining is not convincing. Double staining with dystrophin and spectrin antibodies is very doable. Even if there are some revertant fibers in this mouse model, it is possible to identify donor-derived myobers, if they are existent (thus suggesting, lack of expression of DYS). This reviewer also questions the relevance of finding “4” spectrin positive fibers in dystrophin-deficient mice. When the frequency is so low, it is very possible that similar levels will also be found in the negative control, denoting non-specific binding. This is why it is so important to use multiple antibodies.

In summary, the results involving human engraftment are meaningless because engraftment is very poor (at comparable levels of background staining), and on top of that, the authors are not able to show the rescue of the proper marker (dystrophin) in the recipient DMD mouse model used.

A: As the reviewer mentions, we need to confirm DYSTROPHIN expression following iMuSC transplantation into the diaphragm of DMD model mice. We reanalyzed serial samples of the transplantation shown in Fig. 4 (Fig. R2 below). h-SPECTRIN signals were colocalized with DYSTROPHIN. Moreover, we confirmed h-SPECTRIN was not detected in the negative control (Fig. R3 below) and h-SPECTRIN was an antibody specific for labeling myofibers derived from engrafted iMuSCs. We added an explanation for why we used h-SPECTRIN instead of DYSTROPHIN in the revised manuscript (Page 18, line 298).

Fig.R2 Immunohistochemistry staining 4 weeks after iMuSC transplantation. Sections were stained with anti-DYSTROPHIN (green), anti-h-SPECTRIN antibody (red), and anti-h-NUCLEI antibody (white). Nuclei were stained with DAPI (blue). Scale bars, 20 μm.

Fig. R3 Immunohistochemistry staining 4 weeks after the vehicle transplantation as a negative control. Sections were stained with anti-DYSTROPHIN (green), anti-h-SPECTRIN antibody (red), and anti-h-NUCLEI antibody (white). Nuclei were stained with DAPI (blue). Scale bars, 20 μm.

Minor: line 258 "mouse" should be added prior to “primary cells”.

A: We added “mouse” as suggested in the revised manuscript.
